# Bridging the gap between military prolonged field care monitoring and exploration spaceflight: the compensatory reserve

**DOI:** 10.1038/s41526-019-0089-9

**Published:** 2019-12-04

**Authors:** Taylor E. Schlotman, Kris R. Lehnhardt, Andrew F. Abercromby, Benjamin D. Easter, Meghan E. Downs, L. T. C. Kevin S. Akers, Victor A. Convertino

**Affiliations:** 1 United States Army Institute of Surgical Research 3698 Chambers Pass, Bldg. 3611 JBSA Fort Sam, Houston, TX 78234 USA; 2 NASA Johnson Space Center, 2101 E NASA Pkwy, Houston, TX 77058 USA

**Keywords:** Translational research, Physiology, Biomedical engineering

## Abstract

The concept of prolonged field care (PFC), or medical care applied beyond doctrinal planning timelines, is the top priority capability gap across the US Army. PFC is the idea that combat medics must be prepared to provide medical care to serious casualties in the field without the support of robust medical infrastructure or resources in the event of delayed medical evacuation. With limited resources, significant distances to travel before definitive care, and an inability to evacuate in a timely fashion, medical care during exploration spaceflight constitutes the ultimate example PFC. One of the main capability gaps for PFC in both military and spaceflight settings is the need for technologies for individualized monitoring of a patient’s physiological status. A monitoring capability known as the compensatory reserve measurement (CRM) meets such a requirement. CRM is a small, portable, wearable technology that uses a machine learning and feature extraction-based algorithm to assess real-time changes in hundreds of specific features of arterial waveforms. Future development and advancement of CRM still faces engineering challenges to develop ruggedized wearable sensors that can measure waveforms for determining CRM from multiple sites on the body and account for less than optimal conditions (sweat, water, dirt, blood, movement, etc.). We show here the utility of a military wearable technology, CRM, which can be translated to space exploration.

## Introduction

Major General Barbara R. Holcomb, former Commander of the United States Army Medical Research and Development Command, recently said, “The battlefield of the future is already here and so, as a result, the medical force of the future must be here as well.”^1^ Holcomb was referring to recognition of the concept of prolonged field care (PFC), a top priority across the US Army future medical capability gaps. PFC is field medical care applied beyond doctrinal planning timelines until the patient can be delivered to definitive care.^2^ In other words, combat medics must be prepared to provide medical care to serious casualties in the field with limited resources and without the support of robust medical infrastructure in the event of delayed medical evacuation. The PFC paradigm is a stark contrast to previous battlefield settings where well-developed theaters of war included rapid evacuation to a surgical team at a higher-level of care medical facility. In order to prepare for this change, the U.S. Military is actively researching evolving medical technologies and capabilities to support extended duration patient care in remote and austere environments. Management of serious casualties in such settings will require advancements in training and medical monitoring capabilities for combat medics in the field; this will include improved patient assessment and medical decision making, combined with advanced surgical and medical treatment as well as nursing skills.^2^

With limited resources, significant distances to travel before definitive care, and an inability to evacuate in a timely fashion, medical care in space constitutes the ultimate PFC. During exploration class missions to the Moon or Mars, astronauts will be challenged by delays in real-time telemedicine support from the ground, limited medical supplies due to mass and volume constraints in the spacecraft, and lack of a comprehensively trained medical crew. Successful management of in-flight exploration medical conditions requires development of a care system that prioritizes autonomous operations and promotes appropriate resource utilization.^2^ High-quality, real-time, and predictive monitoring—being able to obtain necessary physiological data points to accurately assess patient status and guide decision making—of casualties would address many of the gaps in PFC for both the military and human spaceflight.^3^ However, the standard traditional clinical approach to assessing patient status relies on measures of vital signs (e.g., heart rate, blood pressure), urine characteristics (e.g., volume, specific gravity, color), blood chemistry (e.g., plasma osmolality), and cognitive functions that tend to stay in normal or near normal ranges during the compensatory phases and provide little information for specific early diagnosis.^4–12^ New innovative technology to assess patient status should provide early, sensitive, and specific indicators of impending physiological compromise. Because physiological compensation is highly individualized, these technologies must also distinguish between various individual responses to better inform accurate medical decision making (i.e., precision medicine).

Under the PFC directive, new technology for individualized monitoring of a patient’s physiological status is one of the key capability gaps listed by the U.S. Military.^2^ Coincidentally, there is a monitoring capability known as the compensatory reserve measurement (CRM) that meets such a requirement. This device monitors physiological status, specifically the cardiovascular system’s ability to compensate for hypovolemia, and provides an indication of impending circulatory collapse. Importantly, CRM analyzes all integrated compensatory mechanisms for blood pressure, systemic blood flow, and tissue oxygenation, specific to both the individual and medical condition.^11^ Real-time monitoring of patient status with CRM has the potential to improve survival not only on future battlefields, but also in space exploration as humans travel farther away from Earth for longer periods of time.

In this paper, we reviewed recent technological advances for clinical monitoring being made in PFC research and how these advances can potentially be used in exploration spaceflight. Specifically, we assessed the successful use of CRM’s sensitive and specific physiologic monitoring capabilities.

## Hypovolemia and physiological compensation

States of hypovolemia can be either absolute, due to reduced circulating blood volume for a given vascular capacitance (e.g., hemorrhage, dehydration), or relative, resulting from expanded vascular capacitance for a given circulating blood volume (e.g., sepsis, heat stress, vasodilation from hypoxia, orthostasis).^10^ In any state, whether absolute, relative, or a combination, hypovolemia causes a cascade of physiological changes, or compensatory mechanisms, that act to maintain blood pressure and adequate tissue oxygenation (e.g., perfusion) in response to the reduction seen at the onset of hypovolemia.^13^ Compensation, a negative feedback mechanism, is dependent upon a complex, inter-related physiological system that begins immediately at the onset of a hypovolemic state as delivery of oxygen (DO_2_) is reduced^14–16^ and arterial and central venous pressures decrease. This, in turn, leads to a reduction in venous return,^10^ impaired baroreflex function,^15,17–20^ decreased cardiac filling pressure, lower stroke volume, and cardiac output,^21^ and increased compliance of the lower extremities,^22,23^ all of which activates both the cardiac (e.g., inotropic, chronotropic) and peripheral vascular (e.g., vasoconstriction) compensatory mechanisms.^10^ Once the body’s capacity to compensate is depleted, the various compensatory mechanisms can no longer maintain hemodynamic stability and a progression towards decompensation (i.e., shock) occurs.

All of these physiological compensatory changes can be visualized in the arterial pressure waveform, which consists of two parts: (1) ejected wave generated when blood moves into the aorta during myocardial contraction and (2) reflected waveform of the blood returning from the peripheral circulation. As such, the features of the ejected waveform reflect all cardiac compensatory mechanisms (e.g., cardiac output and inotropic conditions) and features of the reflected waveform represent compensatory mechanisms that influence peripheral vascular resistance (e.g., tissue perfusion and vascular tone). Thus, all compensatory responses can be represented in a single, integrated signal that can be used to identify failing compensation.^4,11,12,24,25^

The physiological response to central hypovolemia begins with shunting blood to the heart and brain and subsequently progresses to cardiovascular collapse (shock). This response is highly individualized, so identifying those individuals with lower compensatory tolerance to hypovolemia is key to improving survivability. Unfortunately, detecting clinically significant changes in physiological responses to hypovolemia (e.g., orthostatic challenge) usually occurs late in the clinical course of an illness/injury due to the many successful compensatory mechanisms acting within the body to sustain the patient. Current medical capabilities will be challenged on future austere battlefields and exploration space missions that are extending in both time and distance from Earth. These new missions for both the military and human space exploration contexts, highlight the need for the development of advanced monitoring technologies to provide real-time measurements in a compact and portable package. CRM represents a new paradigm that measures the sum total of all mechanisms that compensate for relative blood volume deficit to allow for early recognition of physiological compromise earlier than the development of clinical symptoms.^4,11,26,27^

## Lower-body negative pressure as an experimental tool to induce hypovolemia

**Fig. 1:**
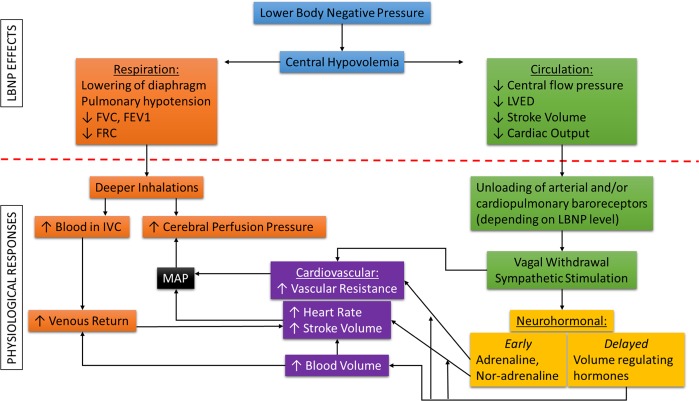
Mechanical effects and physiological responses of lower body negative pressure (LBNP). Modified from.^36^ FVC forced vital capacity, FEV1 forced expiratory volume in 1s, FRC functional residual capacity, LVED left ventricular end-diastolic volume, IVC inferior vena cava, MAP mean arterial pressure.

Lower-body negative pressure (LBNP) provides a unique capability to model acute progressive central hypovolemia (similar to orthostatic hypotension or hemorrhage) in healthy individuals,^28^ by shifting fluid volume from the upper body to the lower body.^29^ It has been demonstrated that the physiological responses to LBNP accurately mimic responses to actual progressive hemorrhage in non-human primates and humans, such that this direct comparison has allowed the equivalent blood loss volume to be estimated during human LBNP experiments.^30^ Importantly, it has been shown repeatedly that LBNP simulates physiological compensatory responses similar to actual blood loss.^31^ The mechanical effects and physiological responses to LBNP are shown in Fig. 1. This research is closely linked with human spaceflight because originally, LBNP was developed by the National Aeronautics and Space Administration (NASA) to model orthostasis. During spaceflight, LBNP has been used since Skylab in the 1970s as an effective way to assess cardiovascular responses in the absence of gravity, providing a technique to evaluate changes in orthostatic tolerance (e.g., hypovolemia).^29^

In our laboratory, LBNP experiments have revealed that two-thirds of the population have high tolerance, or a better ability to compensate for central hypovolemia, while the remaining one-third have low tolerance.^32–34^ This has allowed us to define individuals as either high tolerant (HT) or low tolerant (LT) to central hypovolemia based on tolerance to reduced central blood volume of ~1000 ml (60 mmHg LBNP). HT subjects compensate beyond LBNP ≥ −60 mmHg, while LT subjects reach decompensation at LBNP ≤ −60 mmHg. LBNP has produced repeatable tolerance times (i.e., subjects are either HT or LT)^28,35,36^ and accurately mimics the hemodynamic,^30,31,37–40^ metabolic,^31,41^ hematologic,^31^ ventilatory,^42,43^ neuroendocrine,^31^ and mental status^44^ responses of a hemorrhaging patient. The authors invite the reader to refer to a comprehensive review by Goswami, et al. for a detailed account of LBNP as a tool to investigate the physiology of compensatory responses during conditions of hypovolemia,^36^ as well as a review by Charles et al. for a summary of LBNP use during spaceflight.^29^

## The compensatory reserve

**Fig. 2:**
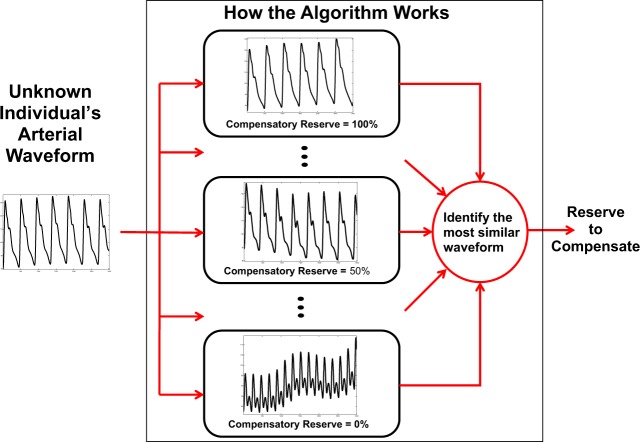
The Compensatory Reserve Measurement (CRM) algorithm framework, from Convertino, et al.^10,11^ Each new individual’s arterial waveform data is compared to the extensive learning library of photoplethysmogram (PPG) arterial waveforms collected on ~200 human subjects undergoing progressive, stepwise lower body negative pressure (LBNP) from baseline (0 mmHg LBNP, 100% CRM) to the point of decompensation (0% CRM). The new waveform is “matched” with the most similar waveform the algorithm has learned from and is assigned a percentage value 0–100% representing the current reserve to compensate for that individual.

From laboratory experiments using LBNP, we have defined the “compensatory reserve” as an individual’s capacity to compensate for reductions in central hypovolemia.^10–12,26,45^ A machine-learning (ML) based algorithm has been developed that provides a CRM, with real-time assessment of an individual’s current physiological status. The CRM is obtained by analyzing changes in photoplethysmogram (PPG) arterial waveforms.^11,12,46^ The sum total of compensatory responses are reflected in observable changes in features of the arterial waveform,^11^ resulting in common identifiable patterns that can be recognized using ML. This ML approach, illustrated in Fig. 2, provides an estimate of the compensatory reserve (e.g., 0–100%, where decompensation occurs at 0%) based on changing features in the arterial waveform of any specific individual, without the need for a baseline value.^11^ The CRM algorithm was developed using a large reference database of arterial waveforms from each individual’s PPG signals, generated from our controlled experiments conducted on over 200 healthy men and women (ages 18–55) during supine rest while subjected to progressive simulated central hypovolemia during LBNP.^24^ The algorithm is not limited to PPG signals for obtaining the arterial waveform; signals from an intra-arterial catheter (i.e. arterial line) can provide an appropriate waveform source to determine CRM values as well. CRM is the first monitoring capability that incorporates and recognizes differences in the full integration of all physiological compensatory mechanisms that protect against inadequate blood pressure regulation, systemic blood flow, and tissue oxygenation associated with hypovolemia.^11,26^ CRM has been compared to measures of tissue oxygenation and validated during hypovolemia in cases of hemorrhage, hyperthermia, dehydration, and physical exertion (described below).

## The compensatory reserve as a reflection of delivery of oxygen

**Fig. 3:**
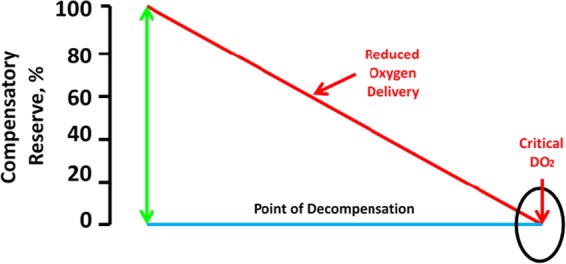
Defining the compensatory reserve. As compensatory reserve is depleted, delivery of oxygen (DO_2_) reduces to critical DO_2_ at the point of decompensation (i.e. 0% compensatory reserve). Modified from.^45^ Moulton, S., Mulligan, J., Grudic, G. Z. & Convertino, V. A. Running on empty? The compensatory reserve index. *J. Trauma Acute Care Surg.* **75**, 1053–1059 (2013).

**Fig. 4:**
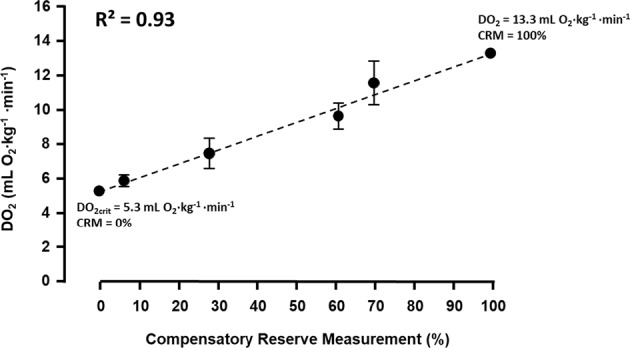
The linear relationship between delivery of oxygen (DO_2_) and compensatory reserve (CRM) can be defined as DO_2_ (mL O_2_ kg^−1^ min^−1^) = 0.08 × CRM (%) + 5.3, as derived from experiments of progressive control hemorrhage and whole blood resuscitation in baboons. From this relationship, the threshold of critical DO_2_ (DO_2crit_) can be defined as 5.3 mL O_2_ kg^−1^ min^−1^. Modified from^47^.

When delivery of oxygen (DO_2_) and/or energy stores in the body’s tissues can no longer meet the energy demand of cells, normal homeostasis is disrupted. Hypovolemia, whether absolute or relative, is a primary physiological contributor to inadequate DO_2_, such that shock occurs when a DO_2_ (DO_2crit_) is reached. An understanding of inadequate DO_2_ and how it relates to early diagnosis and treatment of circulatory shock is important in treating and preventing life-threatening conditions of hypovolemia (i.e. hemorrhage, etc.). In this regard, CRM provides a metric of the integrated compensatory mechanisms that together protect against low tissue perfusion during states of inadequate or compromised DO_2_ and serves to prevent reaching DO_2crit_ (i.e., decompensation with 0% CRM). This concept is illustrated in Fig. 3. As an individual experiences progressive central hypovolemia, a subject progresses along the time course of gradual reduction in DO_2_ towards DO_2crit_, the reserve to compensate is consumed until the point of decompensation, at which the requirements for oxygen at the basic cellular level can no longer be sustained. We demonstrated this concept, shown in Fig. 4, in a study where CRM values were determined for baboons exposed to progressive controlled hemorrhage and whole blood resuscitation.^47^ Once the reserve to compensate is fully depleted (i.e., 0% CRM), DO_2crit_ is reached and hemodynamic decompensation occurs. In a subsequent study, comparing the compensatory responses of women and men during LBNP-induced progressive central hypovolemia, a lower average tolerance observed in the women observed differences was defined by women depleting their reserve to compensate (i.e., CRM) and reaching DO_2crit_ (specifically to the brain) earlier than men.^48^ It is unlikely that this difference is attributed to sex hormones, as menstrual cycle phase was found to be unrelated to LBNP tolerance.^49^

## Measuring compensatory reserve during hypovolemia

### Hemorrhage

**Fig. 5:**
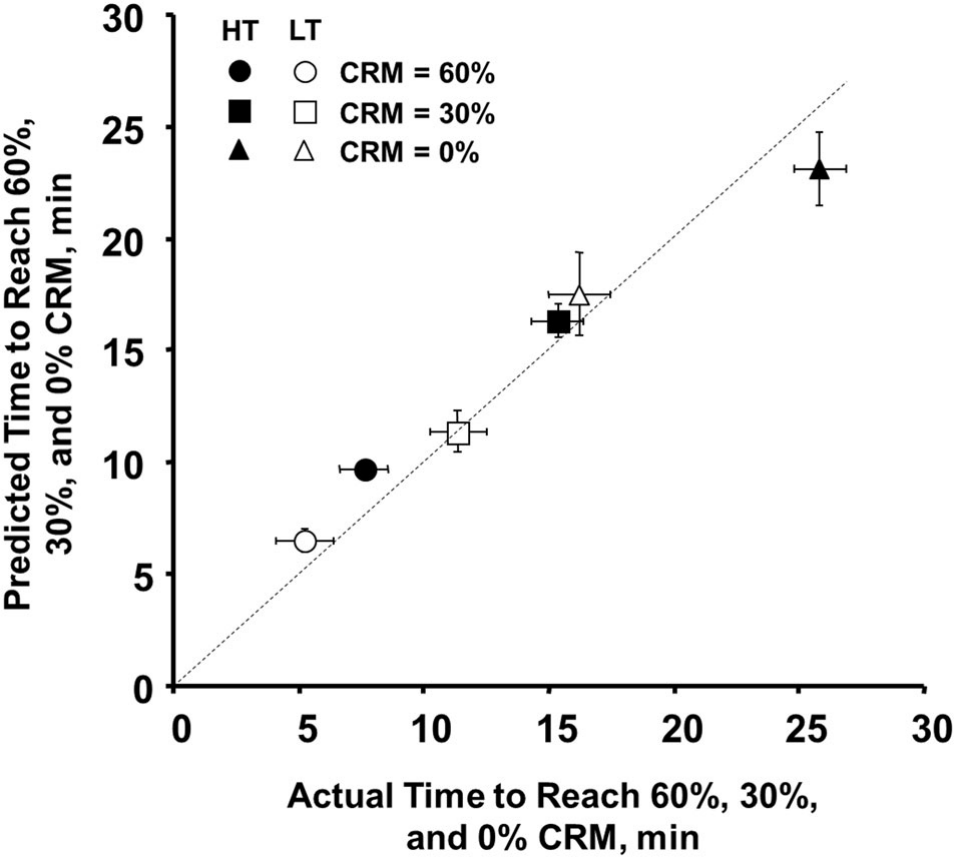
Average and 95% Confidence Interval values of actual and calculated times for compensatory reserve measurements (CRM) at 60% (circles), 30% (squares), and 0% (triangles) CRM in 67 high tolerant (HT) subjects (filled symbols) and 34 low tolerant (LT) subjects (open symbols). Modified from^10,24^.

Using CRM to monitor patient status during hemorrhage, in both experimentally controlled blood withdrawal^12,30,50,51^ and LBNP,^24,30,45^ reveals the advantages of this technology. CRM provides an earlier assessment with higher sensitivity and specificity to predict patient status compared to standard vital signs and other hemodynamic and metabolic measurements. This advantage is demonstrated in Fig. 5, showing a LBNP study with 101 human subjects exposed to progressively stepwise LBNP. Subjects were classified as low tolerant (LT, *n* = 33) or high tolerant (HT, *n* = 68) based on whether they completed (HT) or did not complete (LT) the 60 mmHg LBNP level (an estimated ~1000 ml blood loss) before decompensating.^31^ LT subjects reach 60%, 30%, and 0% CRM significantly faster than HT subjects, and CRM clearly distinguished these two groups. Additionally, the times to reach 60%, 30%, and 0% CRM thresholds for both HT and LT subjects accurately matched the actual recorded times to reach these levels during the experiments. This reaffirms the ability of the CRM algorithm to predict status and outcome, showing that regardless of an individual’s tolerance (HT or LT) to hypovolemia, CRM can accurately predict the time required for each subject to reach specific levels of their reserve to compensate.

**Fig. 6:**
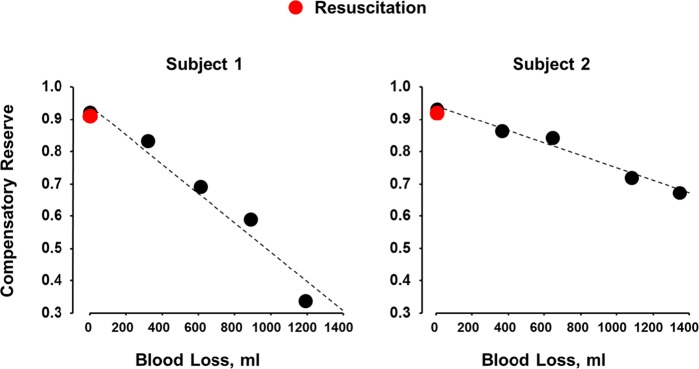
Compensatory reserve (CRM) response during controlled progressive hemorrhage of ~20% circulating blood volume in two healthy volunteers. Subjects had minimal change in standard vital signs but were accurately tracked and differentiated using CRM. The difference in tolerance and variability in compensatory response is highlighted by the difference in slopes between the two subjects. Modified from.^10,12^ Convertino, V. A. & Sawka, M. N. Wearable technology for compensatory reserve to sense hypovolemia. *J. Appl. Physiol.* **124**, 442–451 (2018)^10^ and Convertino, V. A. et al. Individual-specific, beat-to-beat trending of significant human blood loss: the compensatory reserve. *Shock* **44**, 27–32, doi: 10.1097/SHK.0000000000000323 (2015)^12^.

In addition, the CRM algorithm was validated during a study of actual blood loss where twenty healthy subjects underwent experimentally controlled hemorrhage of roughly 20% total circulating blood volume.^12^ CRM displayed significantly greater specificity (0.76) than systolic, diastolic, and mean blood pressures (0.17–0.53), heart rate (0.02), arterial oxygen saturation (0.01), stroke volume (0.33), cardiac output (0.02), and peripheral vascular resistance (0.35). Figure 6 shows clinically relevant relationships related to CRM’s tracking of hypovolemia (i.e., blood loss). Different subjects have different slopes, reinforcing the notion that compensation is highly individual. Importantly, subject 1 used much more of the reserve to compensate (~70%) at approximately 1200 ml blood loss, whereas subject 2 used much less (~30%) despite losing more blood (approximately 1400 ml). These subjects were resuscitated with their own stored blood, returning the subjects’ CRM values to their pre-bleed baseline levels. This highlights the potential of CRM to provide accurate goal-directed resuscitation. Importantly, hemorrhage control and resuscitative efforts may be a critical capability enabling long-duration spaceflight missions outside low Earth orbit. With missions to celestial bodies, the risk of trauma and associated hemorrhage increases: these missions will likely require construction with many extravehicular activities (EVAs) that could result in blunt traumatic injuries or intravehicular hemorrhagic injuries could occur. In partial gravity environments (e.g., approximately 1/6 g on the Moon and 1/3 g on Mars), objects of deceptively lightweight may still have sufficient mass to generate high enough forces to cause traumatic injuries.^52^ It has also been demonstrated that CRM can be used to perform goal-directed resuscitation that promotes good resource utilization. CRM is capable of tracking the maintenance of DO_2_ above shock values (Do_2crit_) to enable optimized resource utilization (i.e. minimal blood or fluid delivered to a patient to maintain adequate levels of DO_2_; Fig. 4 details this relationship). In PFC environments, there will be limited supplies of IV fluids and blood available for transfusion. Therefore, deciding when an individual is sufficiently sick as to need those resources, or has been sufficiently resuscitated to a stable condition, is important.

### Hyperthermia

**Fig. 7:**
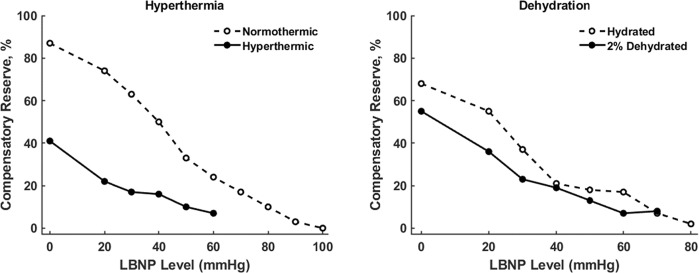
Compensatory reserve (CRM) responses during progressive LBNP to the point of decompensation. *Left*, CRM responses during experimentally controlled normothermic (open circles, dashed) and hyperthermic (filled circles) conditions. *Right*, CRM responses during hydrated (open circles, dashed) and dehydrated conditions (filled circles). Data are presented as means ± 95% confidence intervals. Modified from^10,54^.

Hyperthermia causes cutaneous vasodilation, producing a relative hypovolemic state.^53^ Figure 7 (left panel) presents results from a study in which 12 men were exposed to LBNP in two sessions: (1) normothermia (37 °C core temperature) and (2) hyperthermia (38.2 °C core temperature).^54^ Values of CRM over progressive LBNP were significantly different from the start (0 mmHg LBNP) between normothermic (92% CRM) and hyperthermic (43% CRM) conditions. Additionally, hyperthermic subjects exhausted their total reserve to compensate earlier (−60 mmHg) than normothermic subjects (−100 mmHg). These results indicate CRM has the ability to sense relative hypovolemia resulting from hyperthermia. These data are especially relevant for clinical practice guidelines of both soldiers on battlefields in warmer climates and astronauts with new spacesuits designed that maintain normal body temperatures during EVA activities.

### Dehydration

Dehydration results in absolute hypovolemia by reducing plasma volume, where the magnitude of this reduction is dependent on the type of dehydration and the person’s heat acclimation status.^55^ Figure 7 (right panel) shows results from a study in which hydrated and dehydrated conditions were tested in eight men exposed to LBNP following exercise-heat stress.^54^ During the hydrated test, subjects exercised for 90 min and fluid loss was fully replaced by orally ingesting warm water to maintain normovolemia and euhydration. During the dehydrated test, subjects did not consume any liquid and exercised until the core body temperature increased to the same level observed during the hydrated condition, creating a sweat-induced dehydration. During both tests, subjects were hyperthermic (core temperature 38.2 °C), and, thus, experiencing relative hypovolemia. Hydrated subjects had higher baseline CRM (65% CRM) compared to dehydrated subjects (55% CRM) that translated to a delay in the time required to deplete their reserve to compensate compared to the dehydrated subjects (−70 mmHg vs. −80 mmHg). In these experiments, physiological metrics (e.g., heart rate, blood pressure, body temperature, peripheral vasodilation) were unable to provide precision, sensitivity, and specificity comparable to CRM for predicting the onset of cardiovascular compromise.

### Physical exertion

**Fig. 8:**
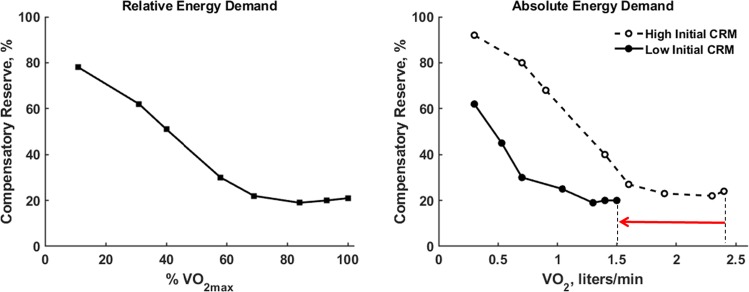
Compensatory reserve (CRM) responses to increases in oxygen consumption (VO_2_) during progressive physical exercise intensity to the point of maximal exertion (VO_2max_). *Left*, relationship between CRM and oxygen requirement as a percentage of maximal effort (%VO_2max_) in 6 subjects. *Right*, lower baseline CRM (filled circles) results in lower VO_2max_ compared to higher baseline CRM (open circles, dashed). Modified from^10^.

The capacity of the cardiovascular system to sustain blood pressure and cardiac output to maintain adequate DO_2_ is a key factor in aerobic performance.^54,56^ The greater oxygen uptake needed during progressive-intensity aerobic exercise leads to greater vasodilation in skeletal muscle (i.e., relative hypovolemia). Figure 8 (left panel) details the relationship between progressively increasing power output until reaching maximal oxygen uptake (VO_2max_) and the compensatory reserve.^10^ The capacity to compensate for increasing energy demand decreased with increasing exercise intensity (%VO_2max_) to an asymptote at ~20% CRM, suggesting that hypovolemia and blood pressure regulation were not the limiting factors during maximal exercise. These results highlight the potential of CRM to guide goal-directed training, such that an individual can gauge evolving fitness or aerobic capacity using CRM during workouts. For example, an individual completing a cycling protocol when starting an exercise regime may require 70% of the compensatory reserve to perform 100 W, leaving only 30%. But after repeated physical training, the same individual may require only 50% compensatory reserve at the same 100 W, indicating an improvement in fitness level (i.e., improved VO_2max_).

Additionally, starting exercise with a higher CRM is associated with a higher VO_2max_ compared to starting with a lower baseline CRM, i.e., lower capacity to compensate for energy demand is associated with limited aerobic capacity [17]. The latter relationship (Fig. 8, right panel) highlights the individual precision provided by CRM, a relationship that is relevant in situations requiring repeated physical exertion that may be required during long-duration missions for both soldiers and astronauts, where an individual may be expected to complete multiple tasks with little recovery time. Also, in the space environment, where exercise is crucial to maintain healthy physical and physiological functions, CRM could be used as a tool to measure astronaut fitness. It has been reported that VO_2max_ can decrease during early spaceflight on the International Space Station (ISS) as much as ~17% before gradually increasing with acclimation.^57^ Though VO_2max_ never returned to preflight values during flight, by recovery day 30 on Earth, astronauts were back to their preflight values.^57^ These results highlight how the use of CRM could monitor how much individuals can handle, how long they can last, how well they are performing, and when they need rest. These results suggest that CRM could monitor minimal requirements (e.g., intensity, duration) for exercise training during and after space missions, with the objective of optimizing task performance during flight and post-flight rehabilitation.

Furthermore, preliminary results indicate CRM tracks a fatigue effect during exercise recovery. Physiologically, the respiratory and skeletal muscle pumps serve as compensatory mechanisms during exercise, reflected in higher CRM values (>30%) even during intense, maximal effort. However, once exercise stops and an individual stops moving, those compensatory pumps stop as well and CRM often decreases suddenly (≤30%) and slowly increases as an individual recovers and returns to baseline physiological status. This tracking may be especially valuable for evaluating readiness in soldiers before and during missions and astronauts before and during EVA. Although additional data are needed to better understand the relationship between the compensatory reserve and physiological responses that can be induced by steady-state exercise of increased intensities and durations (e.g., cardiovascular drift, appearance of blood lactate, increased ventilation volume), it is reasonable to assume that physical exertion performed in spaceflight environments with additional heat loads will compromise the compensatory reserve, as blood pressure regulation is a limiting factor in those environments for aerobic performance.^53^ This is similar to what can be observed during hemorrhage models where bleeding is not stopped.^12,37,46,58–60^

Taken together, data obtained from the studies of human hemorrhage models, physical exercise, heat and dehydration support the notion that CRM may be used to assess compensatory status in other hypovolemic conditions, both absolute and relative. The physiologic condition of hypovolemia is present in each of these studies and the relationship of reduced CRM is reflected with two similar outcomes: (1) the CRM allows assessment of how each condition impacts the capacity to compensate; and (2) lower starting CRM values are associated with earlier exhaustion of the reserve to compensate and, therefore, are predictive of reduced functional outcomes.

### Clinical conditions: trauma & sepsis

There exists preliminary evidence for the usefulness of CRM in clinical settings. Data collected and reported on trauma patients corroborate the use of CRM as an early indicator for improving triage.^61^ In this study, CRM was able to distinguish between a group of 30 patients with blunt trauma and negligible bleeding and a group of 12 patients with penetrating trauma and severe hemorrhage.^11,61^ Blunt trauma patients had an average CRM value around ~60%, while penetrating trauma patients had an average CRM value below ~20%.^11,61^ A prospective observational study of 89 subjects meeting trauma center activation criteria was conducted comparing the predictive capabilities of CRM to those of standard vital signs.^62^ CRM demonstrated superior sensitivity to detect hemorrhage (83% vs. 26%) and negative predictive value (NPV; 91% vs. 78%) compared to SBP; CRM identified hemorrhage requiring lifesaving interventions in the acute phase of injury more reliably than SBP.^62^

**Fig. 9:**
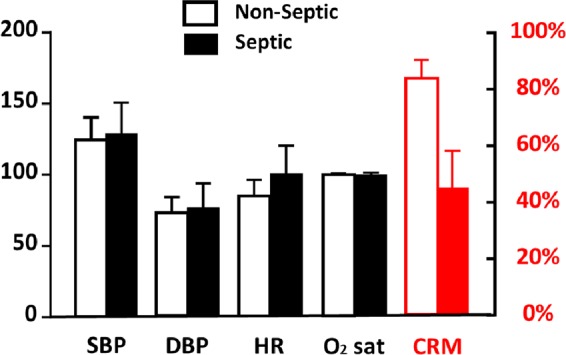
A comparison of standard vital signs and compensatory reserve (CRM) responses in 31 adult patients (18 years and older) who presented with an indication for hospitalization due to a non-traumatic surgical condition, such as appendicitis, diverticulitis, cholangitis, colitis, abscess, etc. from a preliminary analysis. Standard vital signs remain similar between the 25 patients who were verified to be non-septic and the 6 patients with confirmed sepsis, while there is a significant difference in CRM between these groups.

Moreover, a major concern for trauma patients is contracting infection, specifically sepsis. As part of a collaborative effort between the US Army Institute of Surgical Research and the Israeli Defense Force, a preliminary comparison of standard vital signs and CRM was conducted from data collected in 31 adult patients (18 years and older) admitted to the Department of Surgery at Meir Medical Center who presented with an indication for hospitalization due to a non-traumatic surgical condition, such as appendicitis, diverticulitis, cholangitis, colitis, abscess, etc. Patients were excluded if they were pregnant, transferred from other hospitals, prisoners, unable to provide informed consent, or objected to participate at any point during the study. Data were collected prospectively with investigators present from 8am to 5pm on Sunday–Thursday. Noninvasive measurements of CRM were taken 3 times a day and whenever a change was noted in the patient’s hemodynamic status. Patients admitted to the ICU were monitored continually. The comparison between vital signs and CRM showed that while vital signs remain similar between the 25 patients who were verified to be non-septic and the 6 patients with confirmed sepsis, there is a significant difference in CRM between these groups (Fig. 9). These results are especially important for future spaceflight missions as NASA considers trauma to be of the highest level of concern for probable incidence versus impact on both the mission and crew health.^52^

## Design of wearable technology for medical monitoring

One of the major technological challenges in developing and optimizing wearable monitoring technology is providing a sensor that can be placed on the body without interfering with operational activities, is compact and lightweight, and can obtain the desired physiological signal(s) for monitoring. Because of these desired traits, the CRM device uses transmission oximeters to obtain the necessary PPG arterial waveforms to determine CRM values, as these sensors are readily available and in combat medic kits for use on the battlefield. Transmission PPG sensors are commercially available for use on appendages (hands, toes, ears). However, in cases of trauma, appendages may not be available, and current sensors may interfere with military or astronaut activities. Reflectance oximeters provide another option that can be placed in a variety of positions and locations across the body provide a more practical “wear-and-forget” technology. Although these sensors currently have signal-to-noise issues, this type of monitoring technology has become mainstream with smart watches and fitness trackers utilizing these types of sensors. New bio-integrated electronics are also being developed that are small, wireless, water/sweat proof, and skin-like. A version of these new sensors are ultraminiaturized, lightweight, battery-free, and can be implemented for months at a time on hard surfaces such as fingernails and teeth.^63^ Further development of robust PPG technologies could enable an array of sensors to collect information from multiple body sites (e.g., forehead, torso, arms, legs, etc.) simultaneously to determine CRM values. Improvements to sensors may allow for the collection of more waveforms in a wider patient population, and adding to the algorithm’s learning library and advancing its capabilities to the eventual point of being an autonomous technology for monitoring and decision support.

Translating complex physiological signals into usable information to make actionable decisions is one of the most significant human factors challenges in developing wearable technology. In terms of PFC, both for the soldier and astronaut, it is vital to be able to quickly and easily determine a course of action based on the output of the technology. With this in mind, the display for CRM uses a “fuel gauge” approach with “green-yellow-red” indicators, such that the fuel gauge goes from green to yellow at 60% and yellow to red at 30%. Similar to the fuel gauge in an automobile, the display shows a “bar” that reflects the amount of fuel left in the tank (i.e., reserve to compensate). As compensatory mechanisms are working utilized to maintain the gauge changes colors from green (adequate reserve) to yellow (moderate reserve) to red (severely compromised reserve) as a reflection of patient status. Logistical regression of threshold values of CRM associated with hemorrhagic injury in 300 adult trauma patients revealed an optimal lower threshold of 30% CRM (AUC = 0.78) and upper threshold of 60% CRM (AUC = 0.67).^64^ These results validated previously selected thresholds of 30% and 60% CRM.^11,45,51,64^ The trend of patient status is displayed with colored bars showing changes over time. A video showing the responses of a subject undergoing progressive central hypovolemia is available online at https://www.jove.com/video/54737.^46^ The approach using this model accurately estimates compensatory reserve at any point in time with a correlation ≥0.95.^4,24,45^ We have tested the use of CRM in both emergency response teams and combat medics, to gauge if use of CRM reduces time to recognizing a patient is unstable and in need of attention as compared to using standard vital signs alone. In both test groups, addition of the CRM “fuel gauge” significantly reduced time to act by an average of greater than 40%, showing the utility of this metric and design.^60^

## Translation to spaceflight missions

In 2016, NASA Astronaut Scott Kelly returned from a 340-day space mission - the longest ISS mission of any American to date. This mission taught researchers many valuable lessons about what may happen to the human body as we embark on longer duration missions, such as returning to the Moon or the three-year round-trip journey to Mars. Gravity transitions affect spatial orientation, coordination, balance, etc. and residing in prolonged exposure to microgravity presents its own challenges, such as bone and muscle loss, fluid shifts, and dehydration. However, the challenges of this type of mission pale in comparison to exploration missions to the Moon, and more significantly, Mars. These missions will take humans farther away from Earth than ever before, causing significant delays in communications that will demand increased levels of crew member autonomy to provide medical care and respond to emergency situations. The nearly complete Earth independence required of the medical system in exploration spacecraft is the ultimate example of PFC.

Mission performance may be enhanced in future long-duration spaceflight missions by accurately assessing patient status in real-time. Technological challenges faced by military researchers to develop solutions for PFC are very similar to those faced by NASA in preparing for these future missions. Both combat medics and crew medical officers (CMO) in space, while possessing some medical training and skills, are not necessarily physicians and, thus, have a reduced ability to perform resuscitation of critically ill patients in an autonomous fashion. CRM provides a useful tool to monitor both soldier and astronaut status in real time, during training and planning, as well as in the operational environment.

The group at NASA tasked with advancing medical system design and technology development for these missions is the Exploration Medical Capability (ExMC) Element. Among ExMC’s goals is to identify monitoring capabilities for future missions that are easy to use and provide continuous or on-demand remote monitoring of physiological parameters. From NASA’s Human Research Roadmap, two specific knowledge and capability gaps have been identified where the CRM could be applied. Gap 4.18, “limited biomedical monitoring capability for exploration extravehicular activity suits,” and gap 4.19, “we do not have the capability to monitor physiological parameters in a minimally invasive manner during exploration missions,” spell out parameters that a monitoring capability should have; CRM has the ability to fill these gaps. Additionally, ExMC has outlined other medical risk gaps, such as crew training in medical decision-making and real-time decision support (further detail in Table 1), and CRM can apply to a number of these as well. Importantly, CRM devices fit the ideal specifications outlined by NASA, including: (1) meet constraints of spaceflight aimed to minimize mass, volume, and power; (2) ease of operation aimed at shortening start-up time (few seconds), minimizing training requirements, providing wireless store and forward of data, and making for easy integration with spacecraft systems/ data architecture; (3) robust hardware that is battery powered, rechargeable, with long run time between charges (hours to days), sweat proof, long time between failures, easy to troubleshoot and repair; (4) FDA approval or in process for those technologies used for clinical decision support.

**Table 1:** Select Exploration Medical Capabilities (ExMC) medical risk gaps that the CRM may be capable of filling. With more development, it is possible to expand the use and utility of CRM to meet more gaps and help define a concept of operations for medical care.

Med01	ConOps: We do not have a concept of operations for medical care during exploration missions.
Med03	Personalized Medicine: We do not know how we are going to apply personalized medicine to reduce health risk for a selected crew.
Med05	Training for Autonomy: We do not know how to train crew for medical decision making or to perform diagnostic and therapeutic medical procedures to enable extended mission or autonomous operations.
Med07	Real-time Comprehensive Data Processing: We do not have the capability to comprehensively process medically-relevant information to support medical operations during exploration missions.
Med08	Databases and Modeling: We do not have quantified knowledge bases and modeling to estimate medical risk incurred on exploration missions.
Med10	Real-time Decision Support: We do not have the capability to provide computed medical decision support during exploration missions.

In addition to the aforementioned exploration medical gaps, NASA EVA Gap 10 asks “How can knowledge and use of real-time physiological and system parameters during EVA operations improve crew health and performance?” NASA has recommended that heart rate will be used for the purpose of crew health monitoring during exploration EVAs; however, like reported responses in critical care medicine,^26,32,51,65–67^ heart rate has proven to be highly variable among individuals, reducing its accuracy as a sensitive and specific metric of physiological status. However, an interface inside the space suit pressurized volume will be provided to facilitate inclusion of additional biomedical sensing capabilities should they be demonstrated as providing benefit with respect to crew health and/or performance. Furthermore, CRM has the potential to provide a quantifiable and integrated physiological signal during EVA activities for identification of fatigue, heat stress, or fitness for duty, factors of great importance in predicting performance decrements that might lead to catastrophic mistakes.

## Future research for the compensatory reserve

**Fig. 10:**
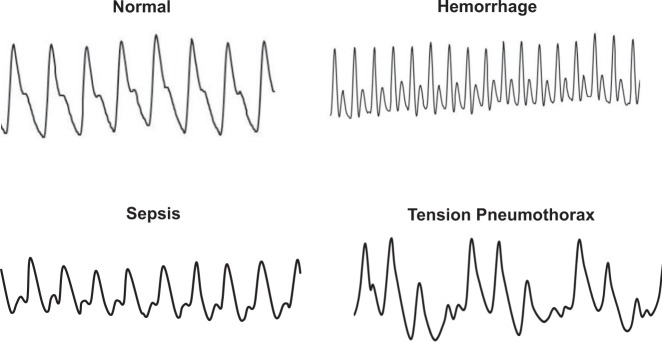
Teaching the CRM algorithm to learn and diagnose clinical conditions. The arterial waveform has a unique signature for different conditions. Thus, teaching the CRM algorithm to recognize those signatures will allow it to become diagnostic in the future.

CRM has emerged as a powerful tool for monitoring by utilizing ML to provide real-time assessment of the current physiological state of individual patients (i.e., precision medicine). Looking to the future of PFC, it will be important to provide medical monitoring technology to help aid in diagnostics to better enable patient care in austere locations. Especially in the case of future battlefields where there is little or no communication availability and missions to Mars with a communications delay of up to 22 minutes in each direction, medical monitoring capabilities are crucial. Expanding the capabilities of the CRM algorithm to be able to not only detect patient compensatory status but also provide diagnostic indicators of specific physiological conditions (e.g., hemorrhage, sepsis, dehydration, etc.) will rely on expanding research to recordings of arterial waveforms from different clinical populations. In order to teach the algorithm to be “smart” enough to recognize different physiological conditions from one another, large quantities of PPG and/or arterial/central line waveforms need to be collected from patients with different conditions to add to the algorithm’s reference library. This approach relies on the basis that each physiological state is phenotypically expressed by an arterial waveform with a unique signature, as shown in Fig. 10, such as hemorrhage, sepsis, tension pneumothorax, and more. Additionally, clinical case studies have been reported that demonstrate the utility of using CRM for medical monitoring in patients with severe hypovolemia from postural orthostatic tachycardia syndrome, childbirth, appendicitis, burn injury, sepsis, cardiopulmonary resuscitation, and Dengue hemorrhagic fever.^67^ Furthermore, the occupational environments for soldiers and astronauts may involve excessively hot conditions that can lead to compromised health, safety, and performance. CRM has the potential to identify those at highest risk for succumbing to heat exhaustion and subsequently, recovery for return to work. Thus, obtaining arterial waveforms for many different clinical and occupational conditions and ‘teaching’ the CRM ML algorithm to recognize those signatures will allow it to become diagnostic in the future. The evidence presented in this paper supports the notion that future development of CRM can be directed to many different applications in both clinical and occupational safety settings.

## Conclusion

Planning, training, and execution of PFC doctrine for long-duration medical care of soldiers on future battlefields and astronauts during extended space exploration missions share similar challenges. We demonstrate here the utility of a wearable technology, CRM, developed for PFC in the military setting that can be translated to space exploration. The identification of reduced CRM in various physiological conditions of absolute or relative hypovolemia reveals that: 1) CRM displays a specificity to detect how different conditions impact individual capacity for cardiovascular compensation; and 2) lower initial CRM is associated with earlier depletion of the compensatory reserve, thus predicting reduced functional outcomes. Our vision is that CRM can be represent a technology that forms a ‘link’ between monitoring soldier and astronaut physiological status in real time, both in training for missions, on the battlefield, or in space. While the initial objective for developing CRM was to monitor the status of combat casualties with severe hemorrhage under PFC conditions, the evidence presented here supports the potential of this technology to be translated to numerous new applications, including exploration spaceflight.

### Disclaimer

The opinions or assertions contained herein are the private views of the authors and are not to be construed as official or as reflecting the views of the Department of the Army, the Department of Defense, or the National Aeronautics and Space Administration.

## References

[1] Khalili, R. A. Prolonged Field Care the New Normal says Army, MRMC Brass. https://mrdc.amedd.army.mil/index.cfm/media/articles/2017/prolonged_field_care_the_new_normal (2017).

[2] Keenan S, Reisberg JC (2017). Prolonged field care: beyond the “Golden Hour”. Wilderness Environ. Med..

[3] Smith M, Withnall R (2018). Developing prolonged field care for contingency operations. Trauma.

[4] Convertino VA (2011). Use of advanced machine-learning techniques for noninvasive monitoring of hemorrhage. J. Trauma.

[5] Cottingham A (2006). Resuscitation of traumatic shock: a hemodynamic review. AACN Adv. Crit. Care.

[6] Orlinsky M, Shoemaker W, Reis ED, Kerstein MD (2001). Current controversies in shock and resuscitation. Surg. Clin. North Am..

[7] Wo C (1993). Unreliability of blood pressure and heart rate to evaluate cardiac output in emergency resuscitation and critical illness. Crit. Care Med..

[8] Bruijns S, Guly H, Bouamra O, Lecky F, Lee W (2013). The value of traditional vital signs, shock index, and age-based markers in predicting trauma mortality. J. Trauma Acute Care Surg..

[9] Parks JK, Elliot AC, Gentilello LM, Shafi S (2006). Systemic hypotension is a late marker of shock after trauma: a validation study of Advanced Trauma Life Support principles in a large national sample. Am. J. Surg..

[10] Convertino VA, Sawka MN (2018). Wearable technology for compensatory reserve to sense hypovolemia. J. Appl. Physiol..

[11] Convertino VA, Wirt MD, Glenn JF, Lein BC (2016). The compensatory reserve for early and accurate prediction of hemodynamic compromise: a review of the underlying physiology. Shock.

[12] Convertino VA (2015). Individual-specific, beat-to-beat trending of significant human blood loss: the compensatory reserve. Shock.

[13] Berne, R. M. & Levy, M. N. *Cardiovascular Physiology*. 6th edn (Mosby-Year Book, Inc., 1992).

[14] Convertino VA (1987). Aerobic fitness, endurance training and orthostatic intolerance. Exerc. Sport Sci. Rev..

[15] Convertino, V. A. in *Handbook of Physiology: Environmental Physiology. III*. *The Gravitational Environment* Vol. 1, Chapter 36 (eds Fregly, M. J. & Blatteis, C. M.) 815-843 (Oxford University Press, 1995).

[16] Ludwig DA, Convertino VA (1994). Predicting orthostatic tolerance: physics or physiology. Aviat. Space Environ. Med.

[17] Convertino VA, Adams WC, Shea JD, Thompson CA, Hoffler GW (1991). Impairment of carotid-cardiac vagal baroreflex in wheelchair dependent paraplegics. Am. J. Physiol. Regul. Integr. Comp. Physiol..

[18] Convertino VA, Doerr DF, Eckberg DL, Fritsch JM, Vernikos-Danellis J (1990). Head-down bed rest impairs vagal baroreflex responses and provokes orthostatic hypotension. J. Appl. Physiol..

[19] Cowley AW, Liard JF, Guyton AC (1973). Role of the baroreceptor reflex in daily control of arterial pressure and other variables in dogs. Circ. Res..

[20] Engelke KA, Doerr DF, Crandall CG, Convertino VA (1996). Application of acute maximal exercise to protect orthostatic tolerance after simulated microgravity. Am. J. Physiol..

[21] Levine B (1993). Regulation of central blood volume and cardiac filling in endurance athletes. The Frank–Starling mechanism as a determinant of orthostatic tolerance. Med Sci. Sports Exerc.

[22] Hoffler, G. W. in *Cardiovascular Flow Dynamics and Measu*reme*nts* (eds N. H. C. Hwang & N. A. Normann) 335-363 (1977).

[23] Luft, U. C., Myhre, L. G., Loeppky, J. A. & Venters, M. D. in *Research Report on Specialized Physiology Studies in Support of Manned Space Flight* 1–60 (Lovelace Foundation, 1976).

[24] Convertino VA, Grudic GZ, Mulligan J, Moulton SL (2013). Estimation of individual-specific progression to impending cardiovascular instability using arterial waveforms. J. Appl Physiol..

[25] Convertino VA (2015). A novel measurement for accurate assessment of clinical status in patients with significant blood loss: the compensatory reserve. Shock.

[26] Convertino VA, Schiller AM (2017). Measuring the compensatory reserve to identify shock. J. Trauma Acute Care Surg..

[27] Convertino VA, Sawka MN (2018). Breathing pattern during and after exercise of different intensities. J. Appl. Physiol..

[28] Convertino VA (2001). Lower body negative pressure as a tool for research in aerospace medicine and military medicine. J. Gravitat Physiol..

[29] Charles J, Lathers C (1994). Summary of lower body negative pressure experiments during space flight. J. Clin. Pharm..

[30] Hinojosa-Laborde C, Howard JT, Mulligan J, Grudic GZ, Convertino VA (2016). Comparison of comensatory reserve during lower-body negative pressure and hemorrhage in nonhuman primates. Am. J. Physiol. Regul. Integr. Comp. Physiol..

[31] Hinojosa-Laborde C (2014). Validation of lower body negative pressure as an experimental model of hemorrhage. J. Appl. Physiol..

[32] Janak JC (2015). Predictors of the onset of hemodynamic decompensation during progressive central hypovolemia: comparison of the peripheral perfusion index, pulse pressure variability, and compensatory reserve index. Shock.

[33] Howard JT, Janak JC, Hinojosa-Laborde C, Convertino VA (2016). Specificity of compensatory reserve and tissue oxygenation as early predictors of tolerance to progressive reductions in central blood volume. Shock.

[34] Schiller AM, Howard JT, Convertino VA (2017). The physiology of blood loss and shock: new insights from a human laboratory model of hemorrhage. Exp. Biol. Med..

[35] Convertino VA, Sather TM (2000). Effects of cholinergic and beta-adrenergic blockade on orthostatic tolerance in healthy subjects. Clin. Auton. Res..

[36] Goswami N, Blaber A, Hinghofer-Szalkay H, Convertino V (2019). Lower body negative pressure: physiological effects, applications, and implementation. Physiol. Rev..

[37] Cooke WH, Ryan KL, Convertino VA (2004). Lower body negative pressure as a model to study progression to acute hemorrhagic shock in humans. J. Appl. Physiol..

[38] Johnson BD (2014). Reductions in central venous pressure by lower body negative pressure or blood loss elicit similar hemodynamic responses. J. Appl. Physiol..

[39] Summers RL (2009). Validation of a computational platform for the analysis of the physiologic mechanisms of a human experimental model of hemorrhage. Resuscitation.

[40] van Helmond N (2015). Coagulation changes during lower body negative pressure and blood loss in humans. Am. J. Physiol. Heart Circ. Physiol..

[41] Ward KR (2010). Oxygen transport characterization of a human model of progressive hemorrhage. Resuscitation.

[42] Convertino VA, Rickards CA, Lurie KG, Ryan KL (2009). Hyperventilation in response to progressive reduction in central blood volume to near syncope. Aviat. Space Environ. Med.

[43] McManus JG (2008). Limitations of end-tidal CO_2_ as an early indicator of central hypovolemia in humans. Prehosp. Emerg. Care.

[44] Ryan KL, Batchinsky A, McManus JG, Rickards CA, Convertino VA (2008). Changes in pulse character and mental status are late responses to central hypovolemia. Prehosp. Emerg. Care.

[45] Moulton S, Mulligan J, Grudic GZ, Convertino VA (2013). Running on empty? The compensatory reserve index. J. Trauma Acute Care Surg..

[46] Convertino, V. A., Hinojosa-Laborde, C., Muniz, G. W. & Carter III, R. Integrated compensatory responses in a human model of hemorrhage. *J. Vis. Exp.* **117**, e54737, 10.3791/54737 (2016). 10.3791/54737 PMC5226259 27911370

[47] Koons, N. J., Nguyen, B., Suresh, M., Hinojosa-Laborde, C. & Convertino, V. A. Tracking DO2 with compensatory reserve during whole blood resuscitation following controlled hemorrhage in baboons. *Shock*. (2019). [Ahead of Print] 10.1097/SHK.0000000000001367. 10.1097/SHK.0000000000001367 32045396

[48] Schlotman TE, Akers KS, Nessen SC, Convertino VA (2019). Differentiating compensatory mechanisms associated to low tolerance to central hypovolemia in females. Am. J. Physiol. Heart Circ. Physiol..

[49] Convertino VA, Schlotman TE, Stacey W, Hinojosa-Laborde C (2019). Tolerance to central hypovolemia is not affected by menstrual cycle phases. Aerosp. Med. Hum. Perform..

[50] Nadler R (2014). The value of non-invasive mesurement of the compensatory reserve index in monitoring and triage of patients experiencing minimal blood loss. Shock.

[51] Stewart CL, Mulligan J, Grudic GZ, Convertino VA, Moulton SL (2014). Detection of low-volume blood loss: the compensatory reserve index versus traditional vital signs. J. Trauma Acute Care Surg..

[52] Kirkpatrick, A. W. et al. Severe traumatic injury during long duration spaceflight: light years beyond ATLS. *J Trauma Manag Outcomes* **3**, 10.1186/1752-2897-3-4 (2009). 10.1186/1752-2897-3-4 PMC2667411 19320976

[53] Nybo, L., Rasmussen, P. & Sawka, M. N. in *Comprehensive Physiology* 93–98 (Wiley, 2014). 10.1002/cphy.c130012 24715563

[54] Gagnon D (2016). The effect of passive heat stress and exercise-induced dehydration on the compensatory reserve during simulated hemorrhage. Shock.

[55] Cheuvront SN, Kenefick RW (2014). Dehydration: physiology, assessment, and performance effects. Compr. Physiol..

[56] Convertino Victor A, Lye Kristen R, Koons Natalie J, Joyner Michael J (2019). Physiological comparison of hemorrhagic shock and V˙O2max: A conceptual framework for defining the limitation of oxygen delivery. Experimental Biology and Medicine.

[57] Moore AD (2014). Peak exercise oxygen uptake during and following long-duration spaceflight. J. Appl. Physiol..

[58] Convertino VA, Rickards CA, Ryan KL (2012). Autonomic mechanisms associated with heart rate and vasoconstrictor reserves. Clin. Auton. Res.

[59] Dinenno FA (2016). Skeletal muscle vasodilation during systemic hypoxia in humans. J. Appl Physiol..

[60] Muniz GW (2013). Promoting early diagnosis of hemodynamic instability during simulated hemorrhage with the use of a real-time decision-assist algorithm. J. Trauma Acute Care Surg..

[61] Stewart CL (2016). The compensatory reserve index following injury: results of a prospective clinical trial. Shock.

[62] Johnson MC (2018). Compensatory Reserve Index: performance of a novel monitoring technology to identify bleeding trauma patients. Shock.

[63] Kim J (2017). Miniaturized battery-free wiresless systems for wearable pulse oximetry. Adv. Funct. Mater..

[64] Wampler MR (2018). Establishing threshold variables for the dashboard view of the compensatory reserve measurement. J. Am. Col. Surg..

[65] Stewart C (2016). The compensatory reserve for early and accurate prediction of hemodynamic compromise: case studies for clinical utility in acute care and physical performance. J. Spec. Oper. Med.

[66] Schiller AM, Howard JT, Lye KR, Magby CG, Convertino VA (2018). Comparisons of traditional metabolic markers and compensatory reserve as early predictors of tolerance to central hypovolemia in humans. Shock.

[67] Moulton SL (2016). State-of-the-art monitoring in dengue shock syndrom: a preliminary report. J. Med Case Rep..

